# Metabolite Profiling of *Dioscorea* (Yam) Leaves to Identify Bioactive Compounds Reveals Their Potential as Renewable Resources

**DOI:** 10.3390/plants10081751

**Published:** 2021-08-23

**Authors:** Min-Ji Kim, Su-Young Son, Su-Gyeong Jeon, Jeong-Gu Kim, Choong-Hwan Lee

**Affiliations:** 1 Department of Bioscience and Biotechnology, Konkuk University, Seoul 05029, Korea; rlaalswl426@naver.com (M.-J.K.); syson119@naver.com (S.-Y.S.); 2 Insititute for Bioresources Research, Gyeongsangbuk-do Agricultural Research and Extension Services, Andong 36614, Korea; grapecorn@korea.kr; 3 Genomics Division, National Academy of Agricultural Science, Rural Development Administration, Jeonju 54874, Korea; 4 Research Institute for Bioactive-Metabolome Network, Konkuk University, Seoul 05029, Korea; 5 MetaMass Co. Ltd., Seoul 05029, Korea

**Keywords:** *Dioscorea*, yam, metabolite profiling, harvest time, antioxidant activity

## Abstract

Yams (*Dioscorea* spp.) are cultivated and consumed as edible tubers, while their leaves are discarded as waste or burned with negative environmental impact. Herein, the metabolites of two yam species (Danma, DAN; Dunggeunma, DUN), harvested in June, July, and August, were profiled using GC-TOF-MS and UHPLC-LTQ-Orbitrap-MS/MS and the antioxidant activity of the extracts was evaluated to stimulate the utilization of yam leaves as a by-product. We observed that the relative levels of amino acids, organic acids, sugars, and saponins decreased linearly with prolonged harvest time, while fatty acid, phenanthrene, and flavonoid levels gradually increased. Furthermore, the leaf extracts obtained in August exhibited the highest antioxidant activity. To determine the antioxidant-contributing metabolites, OPLS-DA was performed for the leaf metabolites of DAN and DUN leaves harvested in August. Hydroxytyrosol-glucoside, apigenin-rhamnoside, and rutin were more abundant in DUN, while luteolin, phenanthrene derivatives, epicatechin, and kaempferide were relatively higher in DAN and their respective metabolites were positively correlated with the antioxidant activity. Moreover, secondary metabolites were more abundant in the leaves than in the roots, and consequently, the antioxidant activity of the former was also higher. Overall, the potential value of yam leaves as a renewable source of bioactive compounds is proposed.

## 1. Introduction

*Dioscorea* (yam) species, comprising approximately 600 variants worldwide, are consumed as an edible root vegetable [1]. Yam species have high nutritional and pharmacological value because they contain polysaccharides, steroidal saponins, allantoin, and polyphenols, such as flavonoids [2]. A number of recent studies have demonstrated that yam roots exhibit various biological and pharmacological activities, such as antioxidant, cholesterol-lowering, growth hormone releasing, and anti-inflammatory, as well as show protective effects against ethanol-induced gastric ulcers [2,3,4]. While yam roots are widely utilized for nutritional and medicinal purposes, yam leaves (the main by-product of yam) are generally burned or discarded as waste, resulting in environmental pollution [5]. In recent years, various studies have investigated the medicinal aspects and chemical compositions of *Dioscorea* roots. However, yam leaves remain relatively little studied, despite showing significant antioxidant and anti-inflammatory activities in vitro and in vivo [6,7]. Although studies on *Dioscorea* species have been focused on the identification of specific metabolites and evaluation of their bioactivity, comprehensive metabolic investigations are limited [8,9].

Nowadays, agricultural and food industry by-products generate large amounts of waste, which negatively impacts the environment [10]. Recently, there has been an increasing interest in reevaluating these residues as sources of food additives or supplements with a high nutritionally added value. Furthermore, Orsat et al. [11] reported that agricultural by-products constitute an abundant resource of bioactive and functional ingredients, including natural antioxidants and antimicrobial compounds, potentially applicable as additives for food, pharmaceutical, and cosmetic products. Several studies have demonstrated the utilization of agricultural by-products, such as pepper leaf (*Capsicum annum* L.) [12], pumpkin leaf (*Telfairia occidentalis* Hook.f.), sweet potato leaf (*Ipomea batatas* L.), cassava leaf (*Manihot esculenta* Crantz) [13], and soybean leaf (*Glycine max* Merr.) [14] as sources of valuable compounds, thereby ameliorating their environmental impact. Therefore, there is considerable incentive to develop value-added products from these waste materials to maximize the utilization of bioactive compounds therein with application in various industries. The manufacturing of such products should ideally be low-cost with minimal environmental impact [10,11].

Mass spectrometry (MS)-based metabolomics is being increasingly employed to characterize complex metabolic changes. Metabolite profiling enables the simultaneous analysis of various metabolites in each sample class without bias [15]. In recent years, plant metabolomics has emerged as an important tool for analyzing natural bioactive products, and is extensively utilized for characterizing functional metabolites and antibiotics as well as plant chemical diversity [16].

In the present study, untargeted metabolite profiling of the leaves of Danma (*Dioscorea ploystchya* Turcz.) and Dunggeunma (*Dioscorea bulbifera* L.), two species of *Dioscorea* widely cultivated in South Korea, was conducted using GC-TOF-MS and UHPLC-LTQ-Orbitrap-MS/MS analysis, and their antioxidant capacity was evaluated. Additionally, the antioxidant activities and metabolite contents were compared among leaves obtained at varying harvest times, as well as among the leaves and roots. The results of this study enabled us to ascertain the potential value of yam leaves for developing high-quality natural ingredients. Thus, we propose yam leaves as an inexpensive, sustainable, and renewable resource of new value-added products applicable in nutraceutical and pharmaceutical industries.

## 2. Results

### 2.1. Differences in the Metabolite Content and Metabolic Pathways in Dioscorea Leaves According to Harvest Time

**Figure 1:**
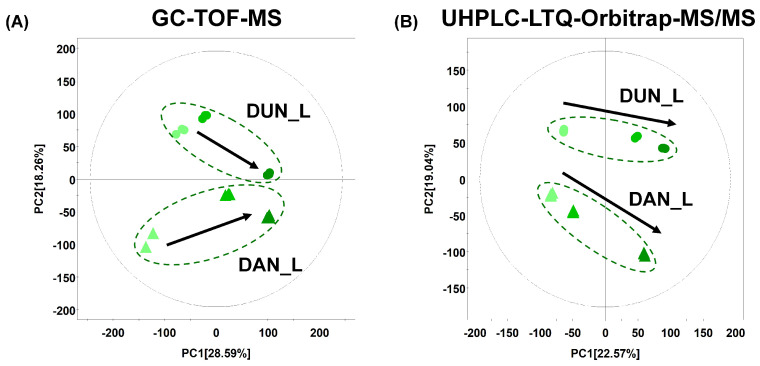
Principle component analysis (PCA) score plots of the metabolites in Danma (DAN) and Dunggeunma (DUN) leaves harvested in June, July, and August, analyzed by (**A**) GC-TOF-MS and (**B**) UHPLC-LTQ-Orbitrap-MS/MS (▲: DAN, ●: DUN, ▲,●, June; ▲,●, July; ▲,●, August).

Primary metabolite profiling of DAN and DUN leaves obtained at different harvest times was performed using GC-TOF-MS combined with multivariate analysis. PCA score plots based on GC-TOF-MS (Figure 1A) showed distinct patterns associated with the harvest time in DUN and DAN leaves across PC1 (28.59%) and each species was discriminated by PC2 (18.26%). PLS-DA with model values of R^2^X (0.733), R^2^Y (0.997), and Q^2^ (0.891) indicated that the fitness and prediction accuracy of the model were similar to the PCA results (Figure S1A). The quality of the model was evaluated by cross-validation analysis (*p* = 6.91 × 10^−5^). Significantly different metabolites of DUN and DAN leaves according to the harvest time were selected based on variable importance in the projection value (VIP > 0.7) and the *p*-value (*p* < 0.05). The selected metabolites were subsequently identified by comparison of the obtained mass fragment patterns with those in the NIST library, Wiley 9th database, and of standard compounds. A total of 40 metabolites were identified, including 10 sugars and sugar alcohols, 8 organic acids, 10 amino acids, 5 fatty acids, 4 miscellaneous metabolites (quinic acid, allantoin, uridine, and adenosine), and 3 unidentified compounds (Table S1).

Moreover, secondary metabolite profiling was performed using UHPLC-LTQ-Orbitrap-MS/MS combined with multivariate analysis. In Figure 1B, the PCA score plots based on UHPLC-LTQ-Orbitrap-MS/MS profiling data in negative ion mode presented distinct metabolomic patterns across harvest time along PC1 (22.57%) components (Figure 1B). Significant separation between DAN and DUN was observed for PC2 (19.04%). A similar pattern for metabolomics data was observed using the PLS-DA score plot for the leaf samples (Figure S1B). The cross-validation values were R^2^X (0.71), R^2^Y (0.999), Q^2^ (0.945), *p* = 6.8 × 10^−6^. A total of 64 different secondary metabolites were tentatively identified (VIP > 0.7, *p* < 0.05) based on various parameters, including their retention time, mass spectra, MS^n^ fragment pattern, elemental composition derived from UHPLC-LTQ-Orbitrap-MS/MS spectra, and comparison with data in published references and web databases. The selected metabolites were identified and divided into sub-classes, with 2 phenanthrenes, 13 flavonoids, 4 steroidal saponins, 16 lipids, 5 miscellaneous metabolites, and 14 unidentified compounds being recognized (Table S2).

**Figure 2:**
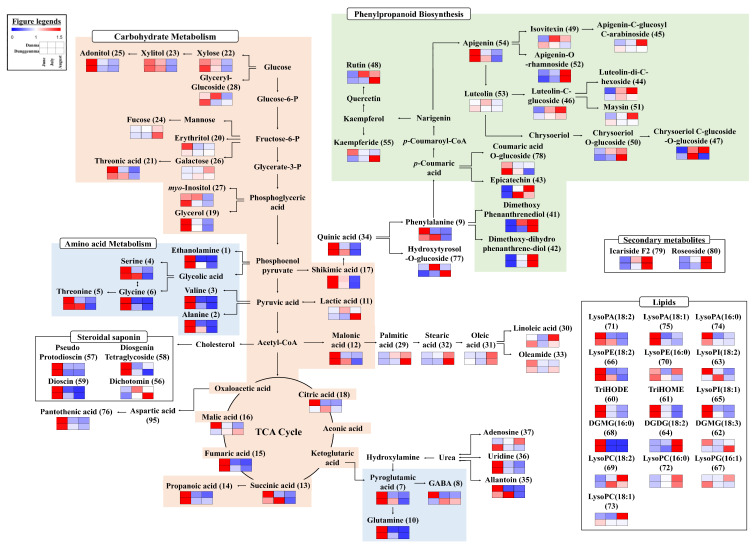
Schematic diagram of primary and secondary metabolic pathways and relative metabolite contents in DAN and DUN leaves harvested in June, July, and August. The pathway was sourced from the KEGG database (http://www.genome.jp/kegg/ accessed on 1 August 2021) and modified. The colored squares (blue-to-red) represent fold changes normalized using the average peak area of each species according to the growth stage.

The selected primary and secondary metabolites were linked to the corresponding metabolic pathways to evaluate their relative contents depending on the growth stage (Figure 2). Each column is expressed as a fold change calculated from the average peak area of each species according to the growth stage. Overall, we observed that the carbohydrate, amino acid, and phenylpropanoid metabolic pathways were appreciably influenced by the growth period. In both leaf types, carbohydrate metabolism, linked to the TCA cycle and amino acid biosynthesis, appeared to decrease during growth, whereas fucose, galactose, lactic acid, malic acid, and GABA levels did not follow this pattern. In contrast, phenylpropanoid metabolism derived from shikimic acid increased during growth. Most of the secondary metabolites exhibited similar patterns during growth, and specific metabolites, including kaempferide, luteolin, and apigenin-C-glucosyl-C-arabinoside showed distinct patterns for DAN and DUN leaves.

### 2.2. Correlation of the Metabolic Profiles and Antioxidant Activities of DAN and DUN Leaves

**Figure 3:**
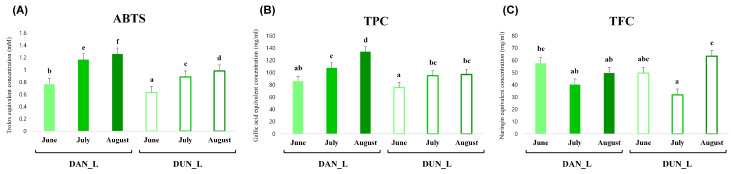
Bioactivities of DAN and DUN leaves obtained at different harvest times (**A**) antioxidant activity (ABTS scavenging capacity), (**B**) total phenolic content (TPC), and (**C**) total flavonoid content (TFC). Values are averages of triplicate measurements (*n* = 3). Each letter represents significantly different values according to Duncan’s multiple-range test (*p* < 0.05).

**Figure 4:**
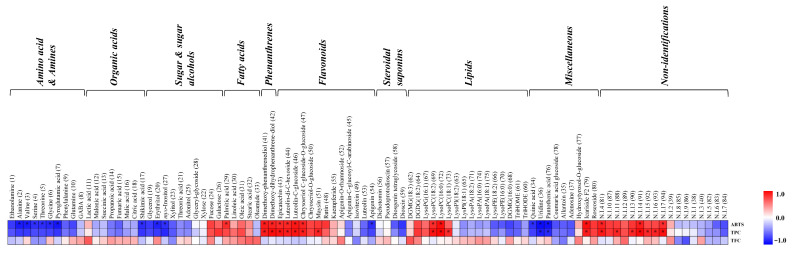
Correlation analysis between relative abundances of significantly discriminant metabolites (VIP > 0.7 and *p* < 0.05) and antioxidant activity (ABTS scavenging capacity), TPC, and TFC of DAN and DUN leaves. Each square indicates “*r*” (Pearson’s correlation coefficient values). Red and blue colors represent positive (0 < *r* < 1) and negative (−1 < *r* < 0) correlations, respectively.

ABTS radical scavenging assays were performed, and total phenolic contents (TPCs) and total flavonoid contents (TFCs) were determined for DUN and DAN leaves collected at different harvest times. The ABTS scavenging capacity and the TPC appeared to be higher for DAN than DUN at each time point; however, the TFC showed a different pattern to that of ABTS scavenging activity and TPC. Intriguingly, the ABTS scavenging activity and TPC gradually increased during growth and were the highest for both species when they were harvested in August (Figure 3). Moreover, we performed correlation analyses between significantly different metabolites and bioactive parameters, namely ABTS scavenging activity, TPC, and TFC (Figure 4). Among them, most of the amino acids, organic acids, sugars and sugar alcohols, lipids, steroidal saponins showed negative correlation, whereas some sugars, such as fucose and galactose, fatty acids, phenanthrenes, and flavonoids displayed positive correlations with the ABTS scavenging activity and TPC. However, the correlation results between metabolites and TFC showed an unclear pattern. In particular, phenanthrenes, flavonoids, lipids, and miscellaneous metabolites, including dimethoxy-phenanthrenediol (41), di-methoxy-dihydrophenanthrenediol (42), epicatechin (43), luteolin-di-C-hexoside (44), luteolin-C-glucoside (46), chrysoeriol C-glucoside-O-glucoside (47), lysoPC (18:2) (69), lysoPC (16:0) (72), and icariside F2 (79) exhibited significant positive correlation with the ABTS scavenging activity and TPC.

**Figure 5:**
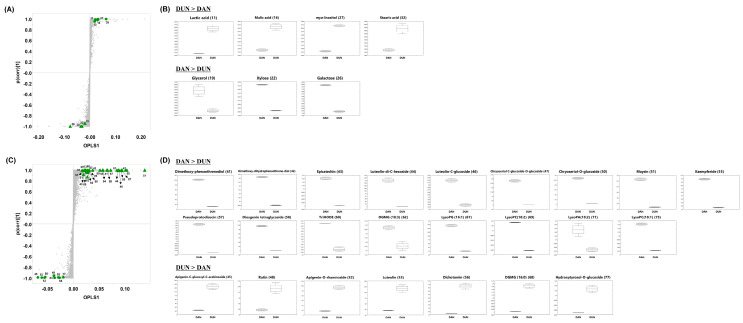
(**A**,**C**) loading S-plots and (**B**,**D**) box and whisker plots showing relative metabolite abundances in DAN and DUN leaves collected in August constructed using (**A**,**B**) GC-TOF-MS and (**C**,**D**) UHPLC-Orbitrap-MS/MS data. Highlighted metabolites (▲: DAN, ●: DUN) in the S-plot indicate statistically significant differences between DAN and DUN leaves collected in August (VIP > 2.5 and *p* < 0.05 in OPLS-DA).

Based on the correlation analysis results, we performed orthogonal partial least square-discriminant analysis (OPLS-DA) on DAN and DUN leaf samples collected in August to determine and validate the metabolites contributing to the antioxidant activity in each leaf type (Figure 3 and Figure S2). OPLS-DA is an extension of PLS-DA, and aims to maximize the explained variance between groups. The significantly discriminant metabolites in DAN and DUN leaves collected in August were determined with clear separation by OPLS component 1, accounting for 85.50% and 83.05% of the variance in the data obtained from GC-TOF-MS and UHPLC-LTQ-Orbitrap-MS/MS analyses, respectively (Figure S2A,B). The OPLS-DA score plot was acquired using *par* scaling to identify metabolite contributions to the difference. Thus, a total of 45 significantly discriminant metabolites (VIP > 2.5, *p*-value < 0.05) were putatively identified between DAN and DUN leaves. Discriminant primary and secondary metabolites are indicated in a loading S-plot (Figure 5A,C) to compare the metabolite content of DAN and DUN leaves. The relative contents of significantly distinctive metabolites in both leaves are visualized in the box and whisker plots shown in Figure 5B,D and Figure S3 A,B. The levels of 3 sugars, 7 flavonoids, 2 steroidal saponins, 2 phenanthrenes, and 6 lipids were significantly higher in DAN than in DUN. On the other hand, 2 organic acids, 1 sugar, 4 flavonoids, 1 steroidal saponin, 1 fatty acid, 1 lipid, and 1 miscellaneous metabolite were more abundant in DUN (Figure 5B,D). The abundances of 22 non-identified compounds are shown in the S-loading plot depicted in Figure S3. Notably among them, 8 metabolites, namely dimethoxy-phenanthrenediol (41), dimethoxy-dihydrophenanthrendiol (42), epicatechin (43), luteolin-di-C-hexoside (44), luteolin C-glucoside (46), chrysoeriol C-glucoside O-glucoside (47), lysoPC (18:2) (71), and lysoPC (16:0) (72) exhibited significantly positive correlation with ABTS scavenging activity. Thus, collectively, these metabolites can be regarded as the major contributors to the potent antioxidant activity and may contribute to the subtle metabolic difference between the DAN and DUN leaves.

### 2.3. Differences between the Antioxidant Activity and Metabolite Composition of the Leaves and Roots

To evaluate the feasibility and value of utilizing the leaves as a by-product, we analyzed the bioactive properties (ABTS scavenging capacity, TPC, and TFC) of August-harvested leaf and root extracts and performed OPLS-DA for comparison. The antioxidant activity of the leaves was higher than that of the roots, irrespective of the species (Figure S4). Similarly, the OPLS-DA model showed a clear separation by OPLS1 (72.00% and 48.46%) between leaf and root samples, according to GC-TOF-MS and UHPLC-LTQ-Orbitrap-MS/MS data, respectively (Figure S5). Based on the OPLS-DA model, 82 discriminant metabolites were identified and drawn as heat maps to compare their relative contents in the leaves and roots (VIP > 0.7, *p*-value < 0.05) (Tables S3 and S4 and Figure S6). Notably, most of the primary metabolites (6 organic acids, 5 amino acids, 3 sugar and sugar alcohols, 5 fatty acids, and 2 miscellaneous metabolites) were more abundant in the roots (Figure S6A). In the case of the relative abundance of secondary metabolites, 2 phenanthrenes, 13 flavonoids, 3 steroidal saponins, 7 lipids, and 4 miscellaneous compounds were found to be present at higher levels in the leaves harvested in August than in the roots (Figure S6B). According to these results, the use of *Dioscorea* leaves as a by-product would be favorable as they display higher antioxidant activity and abundance of certain metabolites compared to the edible root parts.

## 3. Discussion

While numerous recent studies have focused on the analysis of *Dioscorea* roots, to the best of our knowledge, a study comparing the metabolic differences and antioxidant activities of *Dioscorea* leaves has not been conducted [6]. In this study, we conducted untargeted metabolite profiling coupled with bioactivity assays to evaluate the utility of DAN and DUN leaves as value-added product sources. Additionally, we analyzed the spatial metabolic differences between the leaves and roots to delineate plant compound diversity and determine the utility of individual plant parts. These findings suggest the potential value of utilizing *Dioscorea* leaves in various industries.

Harvesting time and plant growth periods are the most crucial factors affecting the metabolite abundance in plants [17,18]. According to our results, the relative contents of metabolites in DAN and DUN leaves differed significantly depending on the harvesting time (Figure 2). Carbohydrate (including sugars, sugar alcohols, and TCA cycle-related metabolites) and amino acid metabolism decreased with prolonged harvest time, whereas phenylpropanoid metabolism increased (Figure 2). In plants, secondary metabolites are produced from the deamination of phenylalanine through the phenylpropanoid pathway and affected by the degree of development at harvest and environmental factors (water availability, salinity, temperature, and light) [19,20,21,22]. Elevated temperatures and sunlight exposure activate the phenylpropanoid biosynthesis pathway resulting in the accumulation of various phenolic compounds due to regulation of enzyme activities, including that of phenylalanine ammonia-lyase (PAL), chalcone synthase (CHS), shikimate dehydrogenase, and polyphenol oxidase [22,23]. Chiara et al. [24] reported that temperature exerts a significant impact on the flavonol concentration in grapevine berry, and the expression of many flavonoid-related genes exhibit drastic temperature-induced fluctuations at the transcription level. Notably, the average seasonal temperature in Korea increases until August [25]. The previous study also reported that the higher exposure to sunlight increased the production of flavonoids in bilberry leaves with high expression of the flavonoid biosynthetic genes such as PAL, CHS, and F3H [26]. In addition, as the leaves used in this study were cultivated in an open field, their metabolite content could be affected by environmental temperature and sunlight to a higher extent than the leaves of plants cultivated in a greenhouse. Furthermore, the total phenolic contents were higher in mature leaves than immature leaves of *Moringa oleifera* [22,27] and of *Hibiscus cannabinus* [28]. Ziaei et al. [29] reported PAL activity of the *Ocimum basilicum* L. leaves, which grown in greenhouse, increased during leaf growth. These were supported by the finding that most of the secondary metabolites, which are related to the phenylpropanoid pathway, were highly distributed in the August samples (Figure 2). In the case of primary metabolism, our results were in agreement with previously reported findings regarding the decrease in carbohydrate and amino acid metabolism in ginseng berry extracts with harvest time (from June to August in Korea) [21]. Generally, the constituents of roots, fruits, leaves, and other green tissues are formed and expand during growth, creating two distinct basic sections, namely “source” tissue (producer and exporter) and “sink” tissue (importer and consumer) [30]. Source tissue includes leaves and other green tissues that produce energy via photosynthesis for plant growth and development, whereas heterotrophic tissues, such as roots and fruits, are sinks [31]. Sugars, organic acids, and amino acids are the major energy sources produced via photosynthesis and respiration in plants [21]. In particular, sugars are important for regulating the source-sink balance and are actively translocated from the source tissue to the sink tissue through phloem [31]. Source-to-sink sugar transport is a major determinant of plant growth [31]. Moreover, amino acids are synthesized from inorganic *N* sources and photosynthates in the leaves. Following the biosynthesis of amino acids, these compounds are released into the cytosol by transporters and transported in the phloem to sink tissues [32]. Furthermore, carbon flux, the basis of plant growth, is distributed into various branches between the primary and secondary metabolic pathways [33]. Up-regulation of the phenylpropanoid biosynthesis pathway, which favors phenolics accumulation, diverts carbon skeletons from primary metabolism into secondary metabolite formation [34]. Considering these factors, we propose that the carbohydrate and amino acid metabolism in yam leaves was affected by the mechanism via which nutrients produced through photosynthesis are transported from source tissues (leaves) to sink tissues (roots) for storage and utilization during formation and development. Moreover, it can be affected by the results of the activation of the phenylpropanoid metabolism together with those metabolic pathways involved in the cleavage of the substantial amounts of carbohydrates.

In this study, we determined the correlation between the in vitro antioxidant activities and metabolites of DAN and DUN leaves. Figure 3 shows that the highest antioxidant activities were observed for August extracts of both species and that DAN leaves exhibited a higher antioxidant activity than DUN leaves. This result revealed that the antioxidant activity of DAN and DUN leaves was due to distinct metabolites. Kyung-Mi et al. [35] reported that DAN roots showed a higher antioxidant activity than DUN roots, but the major metabolites contributing to antioxidant activity were not identified. Therefore, we examined discriminant metabolites present in DAN and DUN leaves collected in August, and found that they were positively correlated with the antioxidant activity and total phenolic contents, allowing us to postulate putative antioxidant compounds. Nowadays, it is well known that phenolic compounds are considered to be the most important antioxidants and are widely distributed among various plant species [36]. Previous study reported that the total antioxidant capacity values including DPPH, FRAP, and ABTS scavenging activity follow the same order as that of phenolic contents in different varieties *Lantana camara* leaves and, in particular, showed that a strong correlation between total phenolic contents with ABTS activity [37]. Similarly, we observed a significant positive correlation (r = 0.946) between total phenolic contents and ABTS activities of yam leaves. Additionally, Lee et al. [38] reported that phenolic compounds including ethyl gallate and quercetin-3-O-glucuronoide, which were detected in the high antioxidant fraction of *A.firma*, showed high ABTS radical scavenging activity and cytoprotective effect against hydrogen peroxide-mediated cytotoxicity. Our results indicated that hydroxytyrosol-O-glucoside, apigenin-O-rhamnoside, and rutin putatively contributed to the antioxidant activity of DUN leaves, whereas epicatechin, luteolin derivatives, kaempferide, and phenanthrene derivatives putatively contributed to the antioxidant activity of DAN leaves. According to recent studies, hydroxytyrosol-O-glucoside, a phenol antioxidant and major chemical constituent of olive leaves, exhibits free radical scavenging activity [39]. The antioxidant activities of the eight flavonoids, epicatechin, rutin, kaempferide, apigenin-O-rhamnoside, and luteolin derivatives have been reported in previous studies [40,41,42,43]. Generally, flavonoids are considered as antioxidants due to their capacity to scavenge free radicals, inhibit lipid oxidation, or chelate metal ions [44]. While the high antioxidant activity of flavonoids is principally attributed to the C2-C3 double bond with a 4-oxo group of the C-ring, which is able to delocalize electrons from the B-ring, it is also affected by the number of hydroxyl groups on the B-ring [43]. Ji Sun et al. [45] suggested that 2,7-dihydroxy-4, 6-dimethoxy phenanthrene, a phenanthrene derivative isolated from yam peel extract, could induce antioxidant enzymes through the Nrf2/ARE-signaling pathway and exert strong antioxidant activity. Thus, we propose that the above-listed metabolites are the major putative contributors to the antioxidant activity of DAN and DUN leaves, respectively.

Because the metabolites and bioactivity of distinct plant parts differ, a single plant can be utilized for various purposes [46]. However, in the case of *Dioscorea*, there are few reports detailing such differences between the edible roots and the largely overlooked leaves. Therefore, we established that there is a significant difference between the metabolite content and antioxidant activity of the leaves and roots to reveal the value of utilizing the leaves. Figure S4 shows the antioxidant activities of the leaves and roots, wherein it can be seen that the leaves exhibited higher overall ABTS scavenging capacity and TPC than the roots, irrespective of the species. Moreover, we identified discriminant metabolites present in the leaves and roots (Figure S6). Consistent with our results, Han et al. [46] reported a higher secondary metabolite content in the above-ground parts of *Zingiber* species than contained in the roots. A possible explanation for this is that the aboveground parts are where the density and intensity of light are focused [46]. However, unlike our findings, the majority of primary metabolites were more abundant in the leaves of *Zingiber* species than in the roots. One possible reason for this difference is that the *Dioscorea* root is an underground storage organ, wherein the synthesis and degradation of starch occur. The derived metabolites are used and stored in the roots as sources of carbon and energy [47,48]. Among the discriminant metabolites in the leaves and roots, the abundance of putative antioxidant metabolites, including hydroxytyrosol-O-glucoside, apigenin-O-rhamnoside, rutin, epicatechin, luteolin derivatives, kaempferide, and phenanthrene derivatives, were relatively higher in the leaf extracts than in the roots of DAN and DUN samples harvested in August (Figures S4 and S5). These results support that the significantly discriminant metabolites, positively correlated with the antioxidant activity of DAN and DUN leaves collected in August, contribute to the high bioactivity of each species. Overall, our findings indicated that *Dioscorea* leaves represent a promising bioresource for various applications.

## 4. Materials and Methods

### 4.1. Chemicals and Reagents

HPLC-grade methanol, water, and acetonitrile were purchased from Fisher Scientific (Pittsburgh, PA, USA). Analytical-grade potassium persulfate, 2,2-azino-bis(3-ethylbenzothiazoline-6-sulfonic acid) diammonium salt (ABTS), Folin–Ciocalteu’s phenol reagent, formic acid, *N*-methyl-*N*-(trimethylsilyl) trifluor-oacetamide (MSTFA), methoxyamine hydrochloride, pyridine, standard compounds (HPLC-grade), 6-hydroxy-2,5,7,8-tetramethylchroman-2-carboxylic acid (Trolox), gallic acid, and naringin were purchased from Sigma-Aldrich (St. Louis, MO, USA). Sodium carbonate and diethylene glycol were obtained from Junsei Chemical Co., Ltd. (Tokyo, Japan).

### 4.2. Plant Material Source and Preparation

Two *Dioscorea* species (Danma and Dunggeunma) were used in this study. All of the leaf and root samples were procured by the Gyeongsangbuk-do Agricultural Research and Extension Services in Andong, South Korea. Both species were cultivated in an open field. Detailed information regarding the harvest date and abbreviation of the corresponding samples is listed in Table 1. All samples were freeze-dried and then ground into a fine powder using a mortar and pestle. The samples were stored at <−70 °C until metabolite extraction and analysis.

### 4.3. Sample Extraction

Each dried powdered sample (100 mg) was extracted with 1 mL of 100% MeOH containing 10 µL of 2-chloro-L-phenylalanine (1 mg/mL) as an internal standard using a MM400 mixer mill (Retsch^®^; Haan, Germany) at a frequency of 30 s^−1^ for 10 min, followed by 5 min of sonication. Subsequently, the extracts were centrifuged at 15,000 rpm for 10 min at 4 °C, and the supernatants were filtered through 0.22-µm polytetrafluoroethylene syringe filters (Chromdisc, Daegu, Korea) and completely dried using a speed-vacuum concentrator (Biotron, Seoul, Korea). The dried samples were reconstituted with 100% MeOH to a final concentration of 10,000 ppm (10 mg/mL) to be used in the antioxidant activity assays and analytical measurements.

### 4.4. GC-TOF-MS Analysis

Each sample extract was subjected to two derivatization reactions, following the method described by Lee et al. [49]. For GC-TOF-MS analysis, an aliquot of each sample extract (100 μL) dissolved in 100% methanol was evaporated using a speed vacuum. Each dried extract was oximated using 50 μL methoxymaine hydrochloride (20 mg/mL) in pyridine at 30 °C for 90 min, followed by reaction with 50 μL of the derivatizing agent, *N*-methyl-*N*-trimethylsilyl-trifluoroacetamide (MSTFA) and incubation at 37 °C for 30 min.

GC-TOF-MS analysis was performed using an Agilent 7890A GC system (Agilent Technologies, Palo Alto, CA, USA) equipped with an Agilent 7693 autosampler and a Pegasus HT TOF-MS (Leco Corporation, St. Joseph, MI, USA). An RTX-5MS column (30 m length × 0.25 mm i.d × 0.25 μm particle size, Restek Corp., St. Joseph, MI, USA) was used to separate the metabolites, with helium as the carrier gas at a constant flow rate of 1.5 mL/min. One microliter of each derivatized sample was injected in split mode (10:1). The temperature of the injector and ion source was 250 °C. The column temperature was held at 75 °C for 2 min, then increased to 300 °C at a rate of 15 °C/min, and finally held for 3 min. The detector voltage was 1606.9 V, and the mass range was 50–700 *m*/*z*. Three analytical replicates of each sample were tested. To decrease the effects of systematic errors, the samples were analyzed in random blocks followed by an intermitted quality control (QC) sample comprising pooled blends from each sample extract [50].

### 4.5. UHPLC-LTQ-Orbitrap-MS/MS Analysis

UHPLC-LTQ-Orbitrap-MS/MS analysis was performed using an UHPLC system equipped with a Vanquish binary pump H system (Thermo Fisher Scientific, Waltham, MA, USA) coupled with an auto-sampler and column compartment. Chromatographic separation was performed on a Phenomenex KINETEX^®^ C18 column (100 mm × 2.1 mm, 1.7 um particle size; Torrance, CA, USA) and the injection volume was 5 μL. The column temperature was set to 40 °C, and the flow rate was 0.3 mL/min. The mobile phase consisted of 0.1% *v*/*v* formic acid in water (A) and 0.1% *v*/*v* formic acid in acetonitrile (B). The gradient parameters were set as follows: 5% B was maintained initially for 1 min, followed by a linear increase to 100% B over 9 min, then sustained at 100% B for 1 min, with a gradual decrease to 5% B over 3 min. The total run time was 14 min. The MS data were collected in the range of 100–2000 *m*/*z* using an ion trap mass spectrometer (Thermo Fisher Scientific, Waltham, MA, USA). The probe heater and capillary temperature were set to 300 °C and 350 °C, respectively. The capillary voltage was set to 2.5 kV in negative mode (positive mode, 3.7 kV).

### 4.6. Data Processing and Multivariate Statistical Analysis

The raw data obtained from GC-TOF-MS and UHPLC-LTQ-Orbitrap-MS/MS analysis were converted to netCDF (*.cdf) format using Leco ChromaTOF and Thermo Xcalibur software. Converted CDF data were preprocessed using the MetAlign software package (http://www/metalign.nl, accessed on 10 February 2021) for peak selection, retention time correction, and alignment. Next, the data processing results were exported to a Microsoft Excel file (.xls). Multivariate statistical analysis was performed using SIMCA-P+ 12.0 software (Umetrics, Umea, Sweden) to determine metabolite differences among samples. We performed PCA, PLS-DA, and OPLS-DA. The significantly discriminant metabolites were selected based on the VIP values of the PLS-DA and OPLS-DA models. The selected metabolites were tentatively identified by comparison of their retention times, mass spectra (MS), and MS fragment patterns (*m*/*z*) with the corresponding data of standard compounds analyzed under similar conditions and of various available databases, including published papers [2,9,51,52,53,54,55,56,57,58,59], the National Institute of Standards and Technology (NIST) library (version 2.0, 2011, FairCom, Gaithersburg, MD, USA), the Dictionary of Natural Products (version 16:2, 2007, Chapman and Hall, USA), Willey8, cioCyc Data-base Collection (http://biocyc.org/, accessed on 10 August 2021), and the Human Metabolome Database (HMDB; http://www.hmdb.ca/, accessed on 10 August 2021). Significance (*p* < 0.05) was tested employing the one-way ANOVA and Student’s *t*-test using Predictive Analytics Soft-Ware (PASW) Statistics 18 software (SPSS Inc., Chicago, IL, USA). Box and whisker plots were generated from the relative metabolite peak areas using STATISTICA 7 software (StatSoft Inc., Tulsa, OK, USA).

### 4.7. Determination of Antioxidant Activity and Total Phenolic and Flavonoid Contents

The method described by Son et al. [60] and Lee et al. [61] was used with several modifications to perform the ABTS scavenging assay and determine the total phenolic and flavonoids contents. For the ABTS assay, 7 mM ABTS reagent was dissolved in 2.45 mM potassium persulfate solution. Next, the mixed solution was incubated in a water bath at 60 °C for 20 min and stored at room temperature for 12 h in the dark. The solution was diluted with distilled water to an absorbance of 0.7 ± 0.02 at 750 nm using a microplate reader (Spectra MAX190, Molecular Devices, San José, CA, USA). Each sample (10 µL) was placed into a well of a 96-well plate along with the stock ABTS solution (190 µL). After incubation for 7 min in the dark, the absorbance of each well was measured at 750 nm using a microplate reader. To evaluate the TPC, 0.2 N Folin–Ciocalteu’s phenol reagent (100 µL) was added to 10 µL of each sample in a 96-well plate and incubated at room temperature for 6 min in the dark. Subsequently, 80 µL of 7.5% sodium carbonate solution was added to the mixture, reacted for 60 min at room temperature, and the absorbance was measured at 750 nm. The results are presented as gallic acid equivalents (ppm). To determine the TFC, 10 µL of extracted sample was added to 180 µL of 90% diethylene glycol and 20 µL of 1 N sodium hydroxide, and then reacted at room temperature for 60 min in the dark. The absorbance was then evaluated at 405 nm using a microplate reader, and the results are presented as naringin equivalents (ppm). All experiments were performed in triplicate.

## 5. Conclusions

In this study, we conducted non-targeted metabolite profiling of DAN and DUN leaves and evaluated their antioxidant activity at different growth stages, correlating the activity with discriminant metabolites. Our results indicated that hydroxytyrosol-O-glucoside, apigenin-O-rhamnoside, and rutin putatively contributed to the antioxidant activity in DUN leaves, whereas epicatechin, luteolin derivatives, kaempferide, and phenanthrene derivatives putatively contributed to the antioxidant activity in DAN leaves. Furthermore, we performed metabolite profiling and evaluated the antioxidant activities of the leaves and roots for comparison. It was found that the leaf samples contained higher levels of putative antioxidant compounds and thereby exhibited higher antioxidant activities than the root samples. Overall, we established that the harvest time significantly affected metabolite variation in *Dioscorea* leaves, and consequently, the antioxidant activity, which increased with prolonged harvest time. This study demonstrates the potential value of *Dioscorea* leaves as a renewable source of valuable antioxidant compounds.

## Figures and Tables

**Table 1:** Details of the samples used in this study.

No.	Common Name	Scientific Name	Gene ^a^	Organ Type	Abbreviation	Collection Date
1	Danma	*Dioscorea ploystchya* Turcz	DC17010	leaf	DAN_L	4 June 2020
2						7 July 2020
3						21 August 2020
4	Dunggeunma	*Dioscorea bulbifera*	DS001	leaf	DUN_L	4 June 2020
5						7 July 2020
6						21 August 2020
7	Danma	*Dioscorea ploystchya* Turcz	DC17010	root	DAN_R	21 August 2020
8	Dunggeunma	*Dioscorea bulbifera*	DS001	root	DUN_R	21 August 2020

^a^ genetic abbreviation.

## Data Availability

The data presented in this study are available on request from the corresponding author.

## Supplementary Materials

The following are available online at https://www.mdpi.com/article/10.3390/plants10081751/s1, Figure S1: Partial least-square discriminant analysis (PLS-DA) of metabolites in Danma (DAN) and Dunggeunma (DUN) leaves harvested at different times, analyzed by GC-TOF-MS and UHPLC-Orbitrap-MS/MS, Figure S2: OPLS-DA score plots for metabolites in DAN and DUN leaves collected in August, obtained using GC-TOF-MS and UHPLC-Orbitrap-MS/MS data, Figure S3: Box and whisker plots showing the relative abundances of unidentified compounds in DAN and DUN leaves collected in August, obtained using GC-TOF-MS and UHPLC-Orbitrap-MS/MS data, Figure S4: Bioactivities of DAN and DUN leaf and root extracts, antioxidant activity based on the ABTS radical scavenging capacity, total phenol content (TPC), total flavonoid content (TFC), Figure S5: OPLS-DA score plots for the metabolites in DAN and DUN leaves and roots collected in August, constructed using GC-TOF-MS and UHPLC-Orbitrap-MS/MS data, Figure S6: Heatmap of significantly different primary and secondary metabolites in DAN and DUN leaves and roots derived using GC-TOF-MS and UHPLC-Orbitrap-MS/MS data, Table S1: Discriminative metabolites in DAN and DUN leaves obtained in June, July, and August derived from the PLS-DA model of the GC-TOF-MS data, Table S2: Discriminative metabolites in DAN and DUN leaves collected in June, July, and August, derived from the PLS-DA model of the UHPLC-LTQ-Orbitrap-MS/MS data, Table S3: Discriminative metabolites in the leaf and root extracts of DAN and DUN harvested in August derived from the OPLS-DA model of the GC-TOF-MS data, Table S4: Discriminative metabolites in the leaf and root extracts of DAN and DUN harvested in August derived from the OPLS-DA model of the UHPLC-LTQ-Orbitrap-MS/MS data.
