# Supplementary material for: Metabolite Profiling of Dioscorea (Yam) Leaves to Identify Bioactive Compounds Reveals Their Potential as Renewable Resources
Source: Plants (Basel). 2021 Aug 23;10(8):1751. doi: 10.3390/plants10081751 (PMC8399132; doi:10.3390/plants10081751)
Supplement: Supplementary file 1 [file plants-10-01751-s001.zip › plants-1335890-supplementary/Supplementary information_Dioscorea_210823.pdf]

Supplementary Information

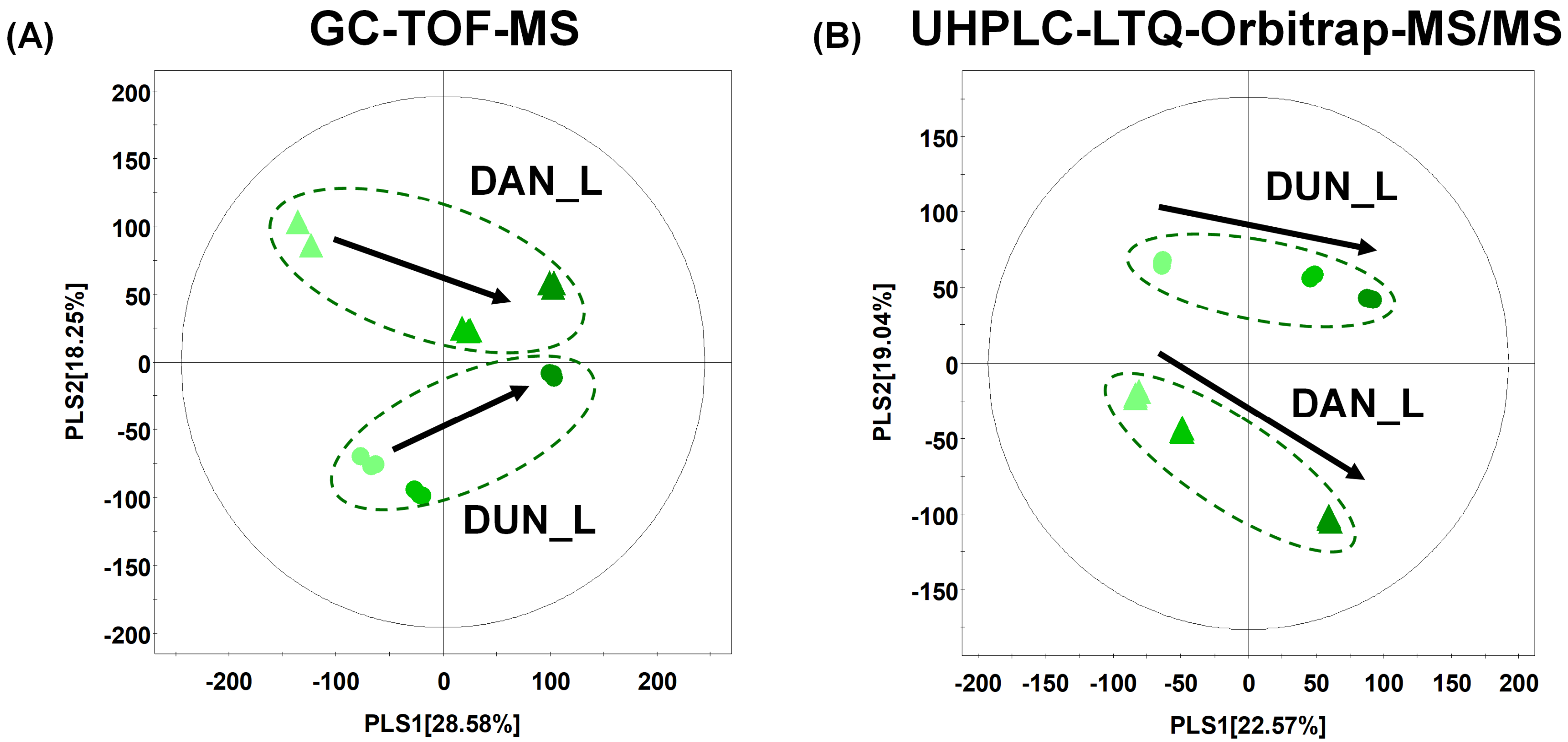

Figure S1. Partial least-square discriminant analysis (PLS-DA) of the metabolites in Danma (DAN) and Dunggeunma (DUN) leaves harvested at different times, analyzed by (A) GC-TOF-MS and (B) UHPLC-Orbitrap-MS/MS (▲: DAN, ●: DUN, ▲, ●, June; ▲, ●, July; ▲, ●, August)

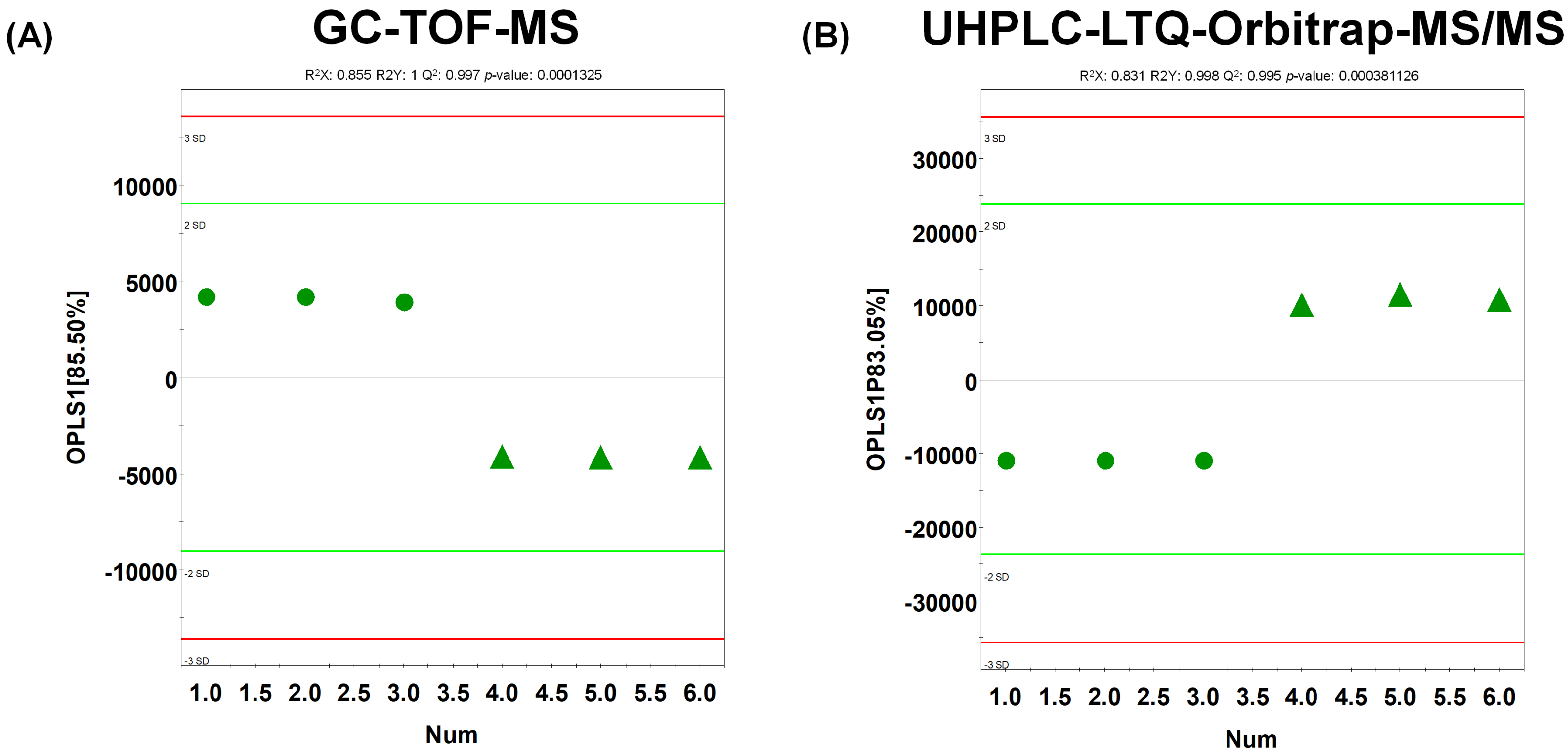

Figure S2. OPLS-DA score plots for metabolites in DAN and DUN leaves collected in August, obtained using (A) GC-TOF-MS and (B) UHPLC-Orbitrap-MS/MS data. (▲: DAN, ●: DUN).

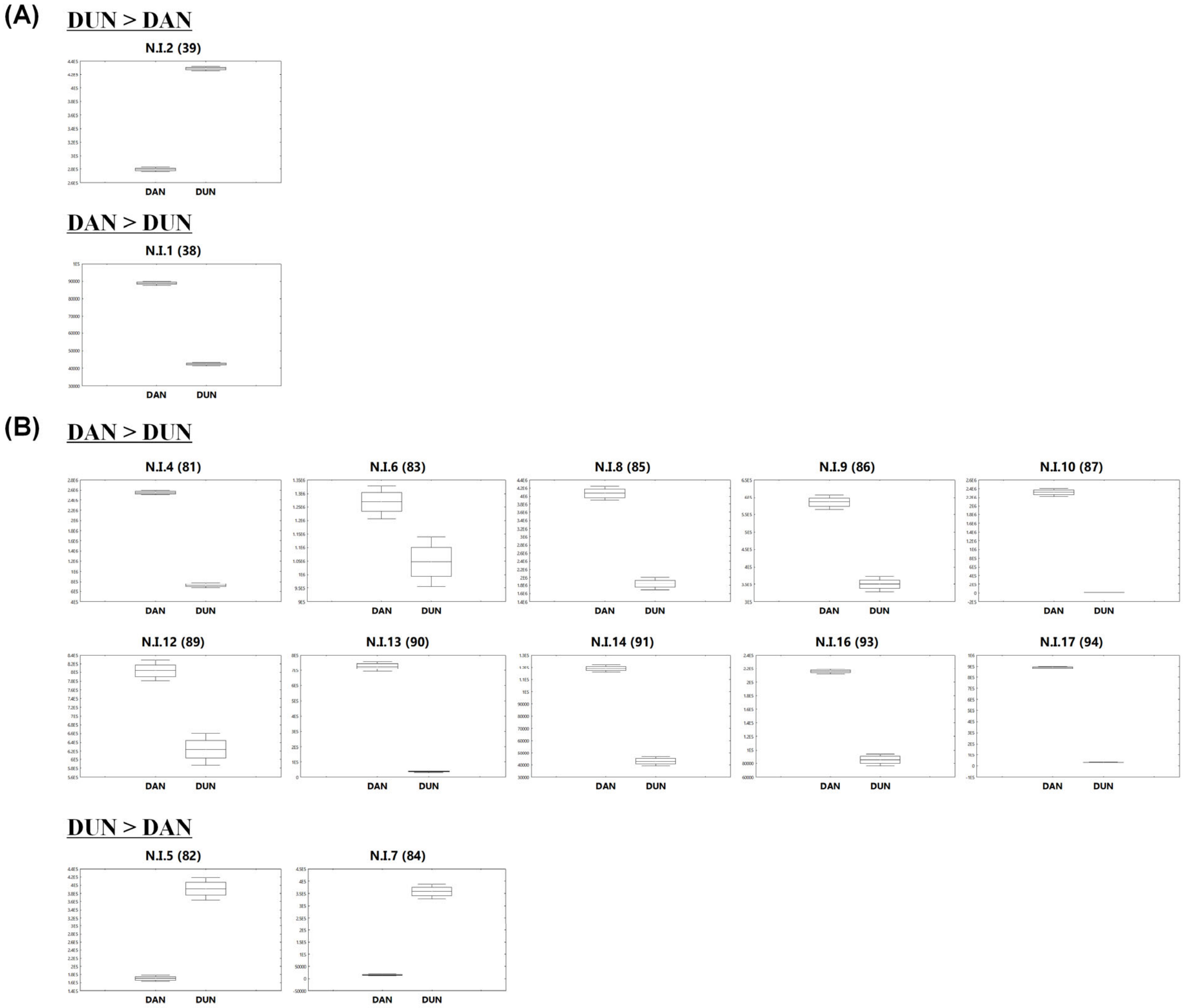

Figure S3. (A, B) Box and whisker plots showing the relative abundances of the non-identified compounds in DAN and DUN leaves collected in August, obtained using (A) GC-TOF-MS and (B) UHPLC-Orbitrap-MS/MS data. (Variable important projection (VIP) value > 2.5 and $p < 0.05$ in OPLS-DA).

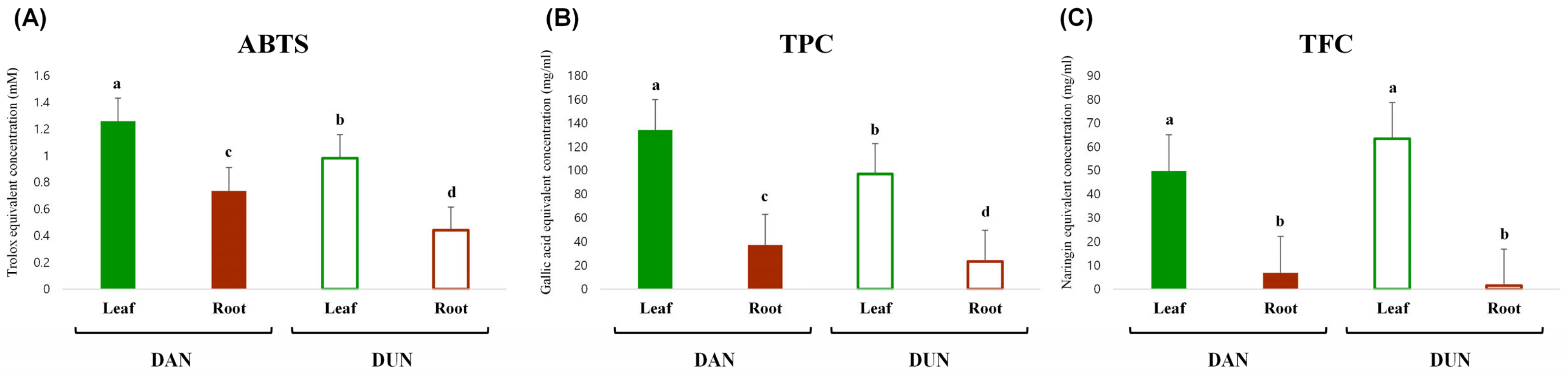

Figure S4. Bioactivities of DAN and DUN leaf and root extracts; (A) antioxidant activity based on ABTS radical scavenging capacity, (B) total phenol content (TPC), and (C) total flavonoid content (TFC). Values are averages of triplicate measurements (n=3). Different letters are significantly different according to Duncan's multiple-range test ($p < 0.05$).

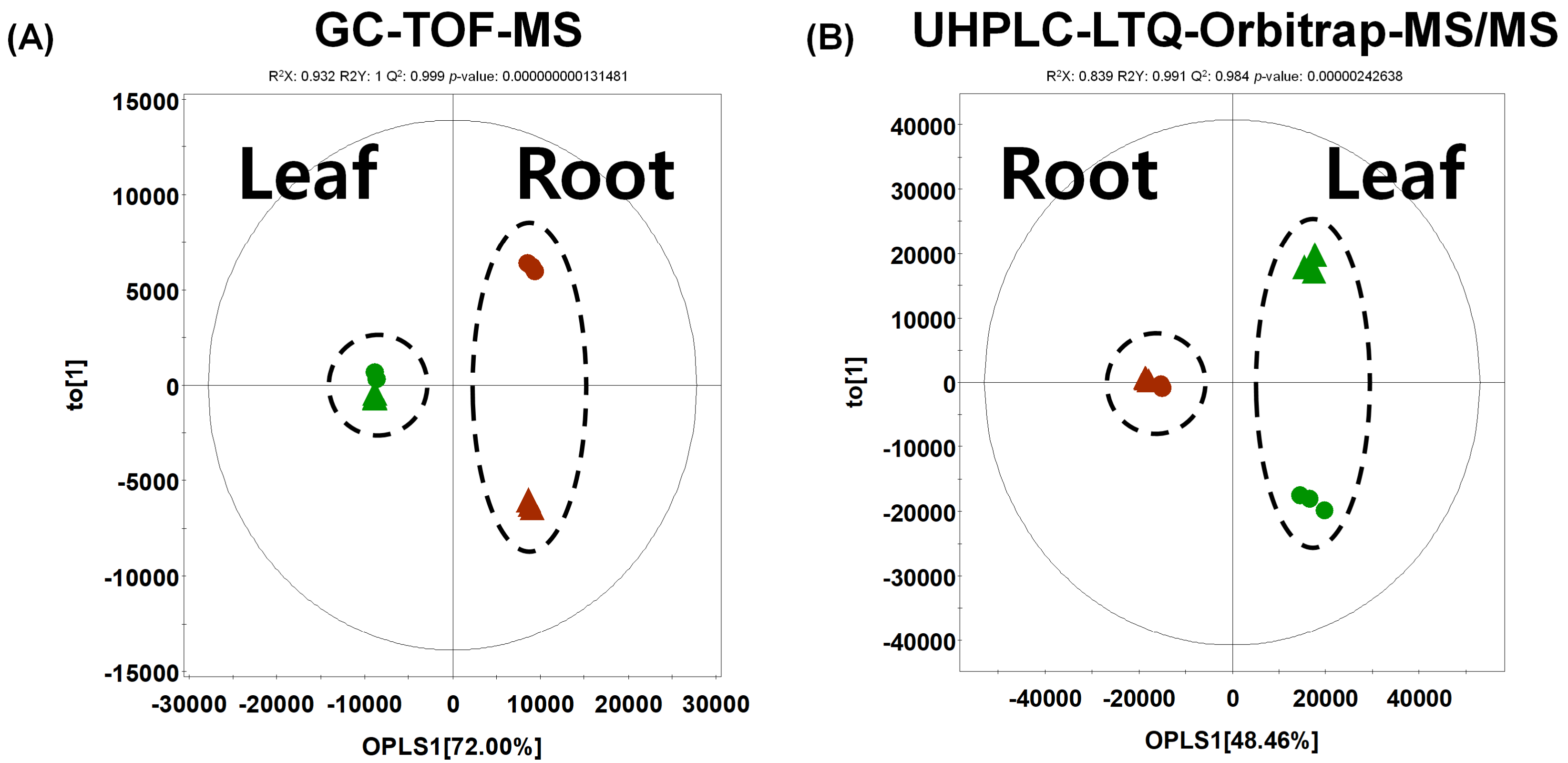

Figure S5. OPLS-DA score plots for the metabolites in DAN and DUN leaves and roots collected in August, constructed using (A) GC-TOF-MS and (B) UHPLC-Orbitrap-MS/MS data. (▲, DAN, leaf, ●: DUN, leaf, ▲: DAN, root, ●: DUN, root).

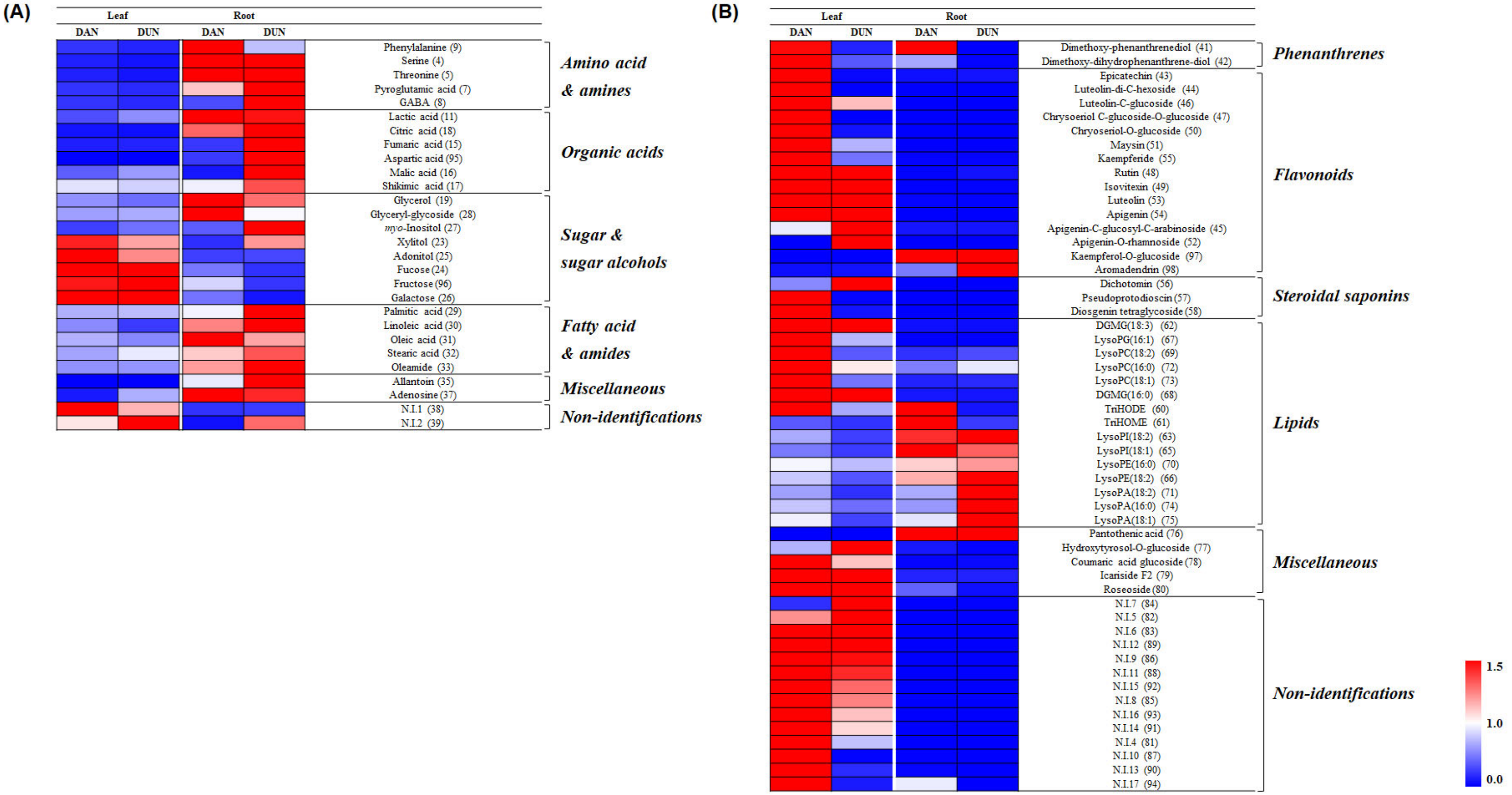

Figure S6. Heatmap of significantly different primary and secondary metabolites in DAN and DUN leaves and roots derived using (A) GC-TOF-MS and (B) UHPLC-Orbitrap-MS/MS data .

Table S1. Discriminative metabolites in DAN and DUN leaves obtained in June, July, and August derived from the PLS-DA model of the GC-TOF-MS data.

No.	Metabolites[a]	Unique Mass	RT[b](min)	MS fragment pattern (*m*/*z*)	TMS[c]	ID[d]
**GC-TOF-MS**						
Amino acid & amines						
1	Ethanolamine	102	4.56	102, 73, 147, 75, 74, 59, 103, 58, 118	2	MS
2	Alanine	116	5.41	116, 73, 75, 147, 117, 59, 74, 103	2	STD, MS
3	Valine	144	6.63	73, 144, 72, 75, 147, 218, 74, 100, 145	2	MS
4	Serine	116	7.06	73, 116, 132, 75, 57, 74, 103, 144	2	STD, MS
5	Threonine	117	7.43	73, 57, 117, 75, 130, 147, 74, 56, 131	2	STD, MS
6	Glycine	174	7.55	174, 73, 86, 100, 175, 59, 248, 133	3	STD, MS
7	Pyroglutamic acid	156	9.48	73, 156, 75, 84, 147, 157, 74, 56, 158	2	STD, MS
8	GABA	174	9.51	73, 174, 147, 75, 86, 59, 175, 100, 301	3	STD, MS
9	Phenylalanine	120	9.67	73, 120, 75, 146, 147, 130, 103, 91, 117	1	STD, MS
10	Glutamine	156	11.36	73, 75, 156, 147, 74, 218, 155, 59, 133	3	STD, MS
Organic acids						
11	Lactic acid	117	4.95	73, 117, 147, 45, 75, 191, 148, 66, 190	2	STD, MS
12	Malonic acid	233	6.52	147, 73, 75, 148, 45, 66, 149, 233	2	STD
13	Succinic acid	247	7.57	147, 73, 75, 148, 55, 56, 149, 247	2	STD, MS
14	Propanoic acid	189	7.77	73, 147, 189, 103, 102, 75, 13, 117	3	MS
15	Fumaric acid	245	7.86	73, 245, 147, 75, 143, 246, 133, 115	2	STD, MS
16	Malic acid	133	9.16	73, 147, 75, 55, 74, 133, 233, 101, 148	3	STD, MS
17	Shikimic acid	204	11.61	73, 204, 147, 75, 205, 74, 133, 206	4	STD, MS
18	Citric acid	273	11.73	73, 147, 75, 273, 74, 67, 211, 148, 149, 133	4	STD, MS
Sugars & sugar alcohols						
19	Glycerol	117	7.22	73, 147, 117, 103, 205, 75, 133, 59, 148	3	STD, MS
20	Erythritol	217	9.29	73, 147, 103, 217, 75, 117, 205, 74, 133, 189	4	MS
21	Threonic acid	292	9.79	73, 147, 75, 117, 103, 292, 220, 74, 102	4	MS
22	Xylose	103	10.74	73, 103, 147, 217, 75, 74, 117, 59, 189, 307	4	STD, MS

23	Xylitol	217	10.88	73, 103, 147, 217, 75, 129, 74	5	STD, MS
24	Fucose	117	11.05	73, 117, 147, 75, 103, 217, 74, 160, 129, 133	4	STD, MS
25	Adonitol	217	11.09	73, 103, 147, 217, 117, 129, 75, 205, 74, 218	5	STD, MS
26	Galactose	103	12.49	73, 147, 103, 75, 160, 117, 129, 205	5	STD, MS
27	*myo*-Inositol	217	13.59	73, 147, 217, 191, 305, 75, 103, 74, 129, 133	6	STD, MS
28	Glyceryl-glycoside	204	14.87	73, 204, 147, 103, 75, 217, 129, 205, 133	6	MS
Fatty acid & amides						
29	Palmitic acid	117	13.11	73, 75, 117, 132, 129, 55, 145, 131	1	STD, MS
30	Linoleic acid	337	14.13	73, 67, 55, 81, 79, 117, 95, 129, 54, 68	1	MS
31	Oleic acid	117	14.15	73, 75, 117, 55, 129, 67, 81, 69, 96, 145	1	STD, MS
32	Stearic acid	117	14.29	73, 117, 75, 132, 129, 55, 145, 131, 57	1	STD, MS
33	Oleamide	131	15.28	73, 131, 55, 144, 116, 128, 59	1	STD, MS
Miscellaneous						
34	Quinic acid	345	12.07	73, 147, 345, 75, 255, 74, 133, 346	5	STD, MS
35	Allantoin	259	13.47	73, 100, 74, 75, 259, 189, 101, 72, 147, 117	3	MS
36	Uridine	217	15.61	73, 217, 75, 103, 147, 169, 218	3	STD, MS
37	Adenosine	230	16.54	73, 230, 236, 103, 75, 245, 217	4	STD, MS
Non-identifications						
38	N.I.1	204	12.84	73, 204, 75, 191, 147, 217, 103, 74, 205	–	–
39	N.I.2	217	13.20	73, 147, 217, 191, 318, 75, 74, 103, 204	–	–
40	N.I.3	169	16.65	73, 103, 147, 217, 129, 75, 169, 74, 361	–	–

[a] Metabolites selected based on a VIP value of > 0.7 and a p-value < 0.05, based on the PLS-DA model.; [b] Retention time; [c] TMS, trimethylsilyl.; [d] Identification; MS, mass spectrum, confirmed based on the data in the National Institutes of Standards and Technology (NIST) database and in-house libraries; STD, mass spectrum, consistent with that of the standard compound.

Table S2. Discriminative metabolites in DAN and DUN leaves collected in June, July, and August derived from the PLS-DA model of the UHPLC-LTQ-Orbitrap-MS/MS data.

UHPLC-LTQ-Orbitrap-MS/MS									
**No.**	**RT[a] (min)**	**[M–H]⁻**	**[M+H]⁺**	**M.W.[b]**	**MS[n] fragment pattern (m/z)[c]**	**Formula**	**Tentative identification[d]**	**Δ ppm**	**REF[e]**
Phenanthrenes									
41	7.15	269.0819	271.0962	270	254> 239> 211	C16H14O4	Dimethoxy-phenanthrenediol	-0.9	[2]
42	8.27	271.0976	273.1115	272	253> 238> 210	C16H16O4	Dimethoxy-dihydrophenanthrene-diol	0.2	[2]
Flavonoids									
43	4.22	289.0716	291.0858	290	245> 203> 175, 188	C15H14O6	Epicatechin	-0.7	[37,38]
44	4.44	609.1458	611.1598	610	429, 489> 309, 351> 266	C27H30O16	Luteolin-di-C-hexoside	-0.6	[38,39]
45	4.50	563.1404	565.1541	564	473, 443> 383, 353	C26H28O14	Apigenin-C-glucosyl-C-arabinoside	-0.3	[38]
46	4.57	447.0930	449.1074	448	327, 357> 299> 255	C21H20O11	Luteolin-C-glucoside	-0.5	[38]
47	4.77	623.1606	625.1758	624	443> 323> 308	C28H32O16	Chrysoeriol C-glucoside-O-glucoside	-0.3	[39]
48	4.82	609.1458	611.1598	610	301, 591> 271, 178> 226	C27H30O16	Rutin	-0.6	[37,39]
49	4.88	431.0978	433.1116	432	311, 341> 283> 239	C21H20O10	Isovitexin	-1.2	[38]
50	4.98	461.1087	463.1231	462	341, 371> 313> 269	C22H22O11	Chryoseriol-O-glucoside	1.6	[39]
51	5.23	575.1406	577.1537	576	489, 473> 369, 399> 351	C27H28O14	Maysin	-0.1	[38]
52	5.82	415.1036	417.1169	416	311, 341> 283> 239, 162	C21H20O9	Apigenin-O-rhamnoside	0.2	[38]
53	6.01	285.0405	287.0544	286	241, 267, 217> 197, 213> 153	C15H10O6	Luteolin	0.6	[40]
54	6.50	269.0458	271.0594	270	225,151> 181> 139	C15H10O5	Apigenin	1.2	[40]
55	6.58	299.0566	301.0699	300	284> 256> 226	C16H12O6	Kaempferide	1.5	[40]
Steroidal saponins									
56	5.75	1193.5958	-	1192	1047> 901> 755	C57H94O26	Dichotomin	0.6	[6]
57	6.16	1075.5311*	-	1030	1075 > 883> 737	C51H82O21	Pseudoprotodioscin	0.7	[6]
58	7.96	1059.5355*	1037.5269**	1014	1013> 867> 721, 575, 161	C51H82O20	Diosgenin tetraglycoside	0.6	[6]
59	8.11	913.47827*	-	868	867> 721> 575	C45H72O16	Dioscin	-2.2	[6]
Lipids									
60	6.36	327.2179	351.2140**	328	309, 171> 127> 123	C18H32O5	TriHODE	0.7	[42]
61	6.65	329.2333	353.2296**	330	229> 211> 183, 167, 193	C18H34O5	TriHOME	-2.6	[42]

62	7.84	721.3653*	675.3601**	676	675, 397> 235, 305> 161	C33H56O14	DGMG(18:3)	0.7	[42]
63	7.87	595.2882	597.3021	596	315, 279, 241> 222, 152> 205	C27H49O12P	LysoPI(18:2)	0.1	[42]
64	8.19	723.3817*	701.3711**	678	677, 397> 235	C33H58O14	DGDG(18:2)	1.2	[42]
65	8.30	597.3046	599.1898	598	582, 315> 222, 153> 79	C27H51O12P	LysoPI(18:1)	0.7	[42]
66	8.33	476.2775	478.2921	477	279> 261> 243	C23H44NO7P	LysoPE(18:2)	-1.6	[42]
67	8.41	481.2564	505.2543**	482	245> 153> 78, 97	C22H43O9P	LysoPG(16:1)	0.1	[42]
68	8.43	653.3756	677.3710**	654	397> 235, 305> 161	C31H58O14	DGMG(16:0)	0.3	[42]
69	8.55	564.3302*	520.3389	519	504> 279> 261	C26H50NO7P	LysoPC(18:2)	1.8	[42]
70	8.75	452.2775	454.2918	453	255> 237> 80	C21H44NO7P	LysoPE(16:0)	-0.5	[42]
71	8.81	433.2358	435.2499	434	153> 78, 97	C21H39O7P	LysoPA(18:2)	-0.7	[42]
72	9.03	540.3316*	496.3386	495	480> 255> 237	C24H50NO7P	LysoPC(16:0)	-1.0	[42]
73	9.11	566.3467*	522.3543	521	506> 281> 263	C26H52NO7P	LysoPC(18:1)	-2.2	[42]
74	9.30	409.2356*	433.2318**	364	153> 79, 97	C19H39O7P	LysoPA(16:0)	-1.1	[42]
75	9.43	435.2505	437.2650	436	153> 79, 97	C21H41O7P	LysoPA(18:1)	-2.8	[42]
Miscellaneous									
76	1.35	218.1037	220.1175	219	146, 88> 59	C9H17NO5	Pantothenic acid	1.0	HMDB
77	1.47	315.1087	339.10458**	316	153> 123> 95	C14H20O8	Hydroxytyrosol-O-glucoside	0.4	[43]
78	3.60	325.0927	349.0891**	326	163, 145> 119	C15H18O8	Coumaric acid O-glucoside	-0.6	[38]
79	4.01	401.1452	425.1405**	402	269> 160> 100	C18H26O10	Icariside F2	-0.3	[44]
80	4.19	431.19199*	409.1827**	386	385> 223, 153, 205> 138	C19H30O8	Roseoside	-1.6	[37]
Non-identifications									
81	4.95	563.1401	565.1545	564	473> 383, 369> 312, 355	-	N.I.4	-	-
82	5.10	417.0829	419.0961	418	357, 327, 399> 297, 339> 269, 253, 225, 163	-	N.I.5	-	-
83	5.22	547.1453	549.1599	548	383> 293> 249, 174	-	N.I.6	-	-
84	5.28	401.0884	403.1012	402	311> 283> 238	-	N.I.7	-	-
85	5.51	559.1447	561.1591	560	395> 293, 321> 175, 249	-	N.I.8	-	-
86	5.93	557.1292	559.1434	558	308, 393, 455> 280> 252	-	N.I.9	-	-
87	5.93	505.2659*	-	460	459> 399> 297, 381, 255, 235, 101	-	N.I.10	-	-
88	6.46	783.4042*	761.3919**	738	737> 719> 371	-	N.I.11	-	-
89	6.55	399.1091	401.1221	400	295, 337> 251, 174> 223, 207	-	N.I.12	-	-
90	6.65	429.1198	431.1326	430	367>309, 339> 266	-	N.I.13	-	-

91	6.68	591.3036	569.2919**	546	545> 253, 309, 527> 160	-	N.I.14	-	-
92	7.01	797.4170*	775.4072**	752	751, 509> 273, 515>209	-	N.I.15	-	-
93	7.70	811.4339*	789.4230**	766	273, 765, 509> 241> 209	-	N.I.16	-	-
94	8.38	537.1557	539.1685	538	522> 507> 492, 479	-	N.I.17	-	-

[a] Retention time; [b] Molecular weight; [c] MS^n fragment patterns detected in the negative ion mode; [d] Tentative metabolites based on VIP analysis with a cut-off value of 0.7 and p-value < 0.05; [e] Reference; * $[M+HCOO]^-$; ** $[M+Na]^+$

Table S3. Discriminative metabolites in the leaf and root extracts of DAN and DUN harvested in August derived from the OPLS-DA model of the GC-TOF-MS data.

No.	Metabolites[a]	Unique Mass	RT[b](min)	MS fragment pattern (*m*/*z*)	TMS[c]	ID[d]
**GC-TOF-MS**						
Amino acids & amines						
4	Serine	116	7.06	73, 116, 132, 75, 57, 74, 103, 144	2	STD, MS
5	Threonine	117	7.43	73, 57, 117, 75, 130, 147, 74, 56, 131	2	STD, MS
7	Pyroglutamic acid	156	9.48	73, 156, 75, 84, 147, 157, 74, 56, 158	2	STD, MS
8	GABA	174	9.51	73, 174, 147, 75, 86, 59, 175, 100, 301	3	STD, MS
9	Phenylalanine	120	9.67	73, 120, 75, 146, 147, 130, 103, 91, 117	1	STD, MS
Organic acids						
11	Lactic acid	117	4.95	73, 117, 147, 45, 75, 191, 148, 66, 190	2	STD, MS
15	Fumaric acid	245	7.86	73, 245, 147, 75, 143, 246, 133, 115	2	STD, MS
95	Aspartic acid	160	8.59	73, 75, 160, 130, 117, 147, 116	2	STD, MS
16	Malic acid	133	9.16	73, 147, 75, 55, 74, 133, 233, 101, 148	3	STD, MS
17	Shikimic acid	204	11.61	73, 204, 147, 75, 205, 74, 133, 206	4	STD, MS
18	Citric acid	273	11.73	73, 147, 75, 273, 74, 67, 211, 148, 149, 133, 274, 183	4	STD, MS
Sugars & sugar alcohols						
19	Glycerol	117	7.22	73, 147, 117, 103, 205, 75, 133, 59, 148	3	STD, MS
23	Xylitol	217	10.88	73, 103, 147, 217, 75, 129, 74	5	STD, MS
24	Fucose	117	11.05	73, 117, 147, 75, 103, 217, 74, 160, 129, 133	4	STD, MS
25	Adonitol	217	11.09	73, 103, 147, 217, 117, 129, 75, 205, 74, 218	5	STD, MS
96	Fructose	103	12.20	73, 103, 147, 217, 133, 75, 59, 89, 74, 117	5	STD, MS
26	Galactose	103	12.49	73, 147, 103, 75, 160, 117, 129, 205	5	STD, MS
27	*myo*-Inositol	217	13.59	73, 147, 217, 191, 305, 75, 103, 74, 129, 133, 318	6	STD, MS
28	Glyceryl-glycoside	204	14.87	73, 204, 147, 103, 75, 217, 129, 205, 133	6	MS
Fatty acids & amides						
29	Palmitic acid	117	13.11	73, 75, 117, 132, 129, 55, 145, 131	1	STD, MS
30	Linoleic acid	337	14.13	73, 67, 55, 81, 79, 117, 95, 129, 54, 68	1	MS
31	Oleic acid	117	14.15	73, 75, 117, 55, 129, 67, 81, 69, 96, 145, 84, 95, 98	1	STD, MS

32	Stearic acid	117	14.29	73, 117, 75, 132, 129, 55, 145, 131, 57	1	STD, MS
33	Oleamide	131	15.28	73, 131, 55, 144, 116, 128, 59	1	STD, MS
Miscellaneous						
35	Allantoin	259	13.47	73, 100, 74, 75, 259, 189, 101, 72, 147, 117	3	MS
37	Adenosine	230	16.54	73, 230, 236, 103, 75, 245, 217	4	STD, MS
Non-identifications						
38	N.I.1	204	12.84	73, 204, 75, 191, 147, 217, 103, 74, 205	–	–
39	N.I.2	217	13.20	73, 147, 217, 191, 318, 75, 74, 103, 204	–	–

[a] Metabolites selected by VIP value (> 0.7) and a p-value < 0.05 based on PLS-DA model.; [b] Retention time; [c] TMS, trimethylsilyl.; [d] Identification; MS, mass spectrum, confirmed with the National Institutes of Standards and Technology (NIST) database and in-house libraries; STD, mass spectrum, consistent with that of the standard compounds.

Table S4. Discriminative metabolites in the leaf and root extract of DAN and DUN harvested in August derived from the OPLS-DA model of the UHPLC-LTQ-Orbitrap-MS/MS data

UHPLC-LTQ-Orbitrap-MS/MS									
**No.**	**RT[a] (min)**	**[M–H]⁻**	**[M+H]⁺**	**M.W.[b]**	**MS^n fragment pattern (m/z)[c]**	**Formula**	**Tentative Identification[d]**	**Δ ppm**	**REF[e]**
Phenanthrenes									
41	7.15	269.0819	271.0962	270	254> 239> 211	C16H14O4	Dimethoxy-phenanthrenediol	-0.9	[2]
42	8.27	271.0976	273.1115	272	253> 238> 210	C16H16O4	Dimethoxy-dihydrophenanthrene-diol	0.2	[2]
Flavonoids									
43	4.22	289.0716	291.0858	290	245> 203> 175, 188	C15H14O6	Epicatechin	-0.7	[37,38]
44	4.44	609.1458	611.1598	610	429, 489> 309, 351> 266	C27H30O16	Luteolin-di-C-hexoside	-0.6	[38,39]
45	4.50	563.1404	565.1541	564	473, 443> 383, 353	C26H28O14	Apigenin-C-glucosyl-C-arabinoside	-0.3	[38]
46	4.57	447.0930	449.1074	448	327, 357> 299> 255	C21H20O11	Luteolin-C-glucoside	-0.5	[38]
47	4.77	623.1606	625.1758	624	443> 323> 308	C28H32O16	Chrysoeriol C-glucoside-O-glucoside	-0.3	[39]
48	4.82	609.1458	611.1598	610	301, 591> 271, 178> 226	C27H30O16	Rutin	-0.6	[37,39]
49	4.88	431.0978	433.1116	432	311, 341> 283> 239	C21H20O10	Isovitexin	-1.2	[38]
50	4.98	461.1087	463.1231	462	341, 371> 313> 269	C22H22O11	Chryoseriol-O-glucoside	1.6	[39]
97	5.12	447.0930	449.1068	448	284, 327, 357> 255> 227, 211	C21H20O11	Kaempferol-O-glucoside	-0.7	[45]
51	5.23	575.1406	577.1537	576	489, 473> 369, 399> 351	C27H28O14	Maysin	-0.1	[38]
98	5.44	287.0561	289.0700	288	259> 215> 173	C15H12O6	Aromadendrin	0.0	[45]
52	5.82	415.1036	417.1169	416	311, 341> 283> 239, 162	C21H20O9	Apigenin-O-rhamnoside	0.2	[38]
53	6.01	285.0405	287.0544	286	241, 267, 217> 197, 213> 153	C15H10O6	Luteolin	0.6	[40]
54	6.50	269.0458	271.0594	270	225,151> 181> 139	C15H10O5	Apigenin	1.2	[40]
55	6.58	299.0566	301.0699	300	284> 256> 226	C16H12O6	Kaempferide	1.5	[40]
Steroidal saponins									
56	5.75	1193.5958	-	1192	1047> 901> 755	C57H94O26	Dichotomin	0.6	[6]
57	6.16	1075.5311*	-	1030	1075 > 883> 737	C51H82O21	Pseudoprotodioscin	0.7	[6]
58	7.96	1059.5355*	1037.5269**	1014	1013> 867> 721, 575, 161	C51H82O20	Diosgenin tetraglycoside	0.6	[6]
Lipids									
60	6.36	327.2179	351.2140**	328	309, 171> 127> 123	C18H32O5	TriHODE	0.7	[42]
61	6.65	329.2333	353.2296**	330	229> 211> 183, 167, 193	C18H34O5	TriHOME	-2.6	[42]
62	7.84	721.3653*	675.3601**	676	675, 397> 235, 305> 161	C33H56O14	DGMG(18:3)	0.7	[42]

63	7.87	595.2882	597.3021	596	315, 279, 241> 222, 152> 205	C27H49O12P	LysoPI(18:2)	0.1	[42]
65	8.30	597.3046	599.1898	598	582, 315> 222, 153> 79	C27H51O12P	LysoPI(18:1)	0.7	[42]
66	8.33	476.2775	478.2921	477	279> 261> 243	C23H44NO7P	LysoPE(18:2)	-1.6	[42]
67	8.41	481.2564	505.2543**	482	245> 153> 78, 97	C22H43O9P	LysoPG(16:1)	0.1	[42]
68	8.43	653.3756	677.3710**	654	397> 235, 305> 161	C31H58O14	DGMG(16:0)	0.3	[42]
69	8.55	564.3302*	520.3389	519	504> 279> 261	C26H50NO7P	LysoPC(18:2)	1.8	[42]
70	8.75	452.2775	454.2918	453	255> 237> 80	C21H44NO7P	LysoPE(16:0)	-0.5	[42]
71	8.81	433.2358	435.2499	434	153> 78, 97	C21H39O7P	LysoPA(18:2)	-0.7	[42]
72	9.03	540.3316*	496.3386	495	480> 255> 237	C24H50NO7P	LysoPC(16:0)	-1.0	[42]
73	9.11	566.3467*	522.3543	521	506> 281> 263	C26H52NO7P	LysoPC(18:1)	-2.2	[42]
74	9.30	409.2356*	433.2318**	364	153> 79, 97	C19H39O7P	LysoPA(16:0)	-1.1	[42]
75	9.43	435.2505	437.2650	436	153> 79, 97	C21H41O7P	LysoPA(18:1)	-2.8	[42]
Miscellaneous									
76	1.35	218.1037	220.1175	219	146, 88> 59	C9H17NO5	Pantothenic acid	1.0	HMDB
77	1.47	315.1087	339.10458**	316	153> 123> 95	C14H20O8	Hydroxytyrosol-O-glucoside	0.4	[43]
78	3.60	325.0927	349.0891**	326	163, 145> 119	C15H18O8	Coumaric acid glucoside	-0.6	[38]
79	4.01	401.1452	425.1405**	402	269> 160> 100	C18H26O10	Icariside F2	-0.3	[44]
80	4.19	431.19199*	409.18265**	386	385> 223, 153, 205> 138	C19H30O8	Roseoside	-1.6	[37]
Non-identifications									
81	4.95	563.1401	565.1545	564	473> 383, 369> 312, 355	-	N.I.4	-	-
82	5.10	417.0829	419.0961	418	357, 327, 399> 297, 339> 269, 253, 225, 163	-	N.I.5	-	-
83	5.22	547.1453	549.1599	548	383> 293> 249, 174	-	N.I.6	-	-
84	5.28	401.0884	403.1012	402	311> 283> 238	-	N.I.7	-	-
85	5.51	559.1447	561.1591	560	395> 293, 321> 175, 249	-	N.I.8	-	-
86	5.93	557.1292	559.1434	558	308, 393, 455> 280> 252	-	N.I.9	-	-
87	5.93	505.2659*	-	460	459> 399> 297, 381, 255, 235, 101	-	N.I.10	-	-
88	6.46	783.4042*	761.3919**	738	737> 719> 371	-	N.I.11	-	-
89	6.55	399.1091	401.1221	400	295, 337> 251, 174> 223, 207	-	N.I.12	-	-
90	6.65	429.1198	431.1326	430	367>309, 339> 266	-	N.I.13	-	-
91	6.68	591.3036	569.2919**	546	545> 253, 309, 527> 160	-	N.I.14	-	-
92	7.01	797.4170*	775.4072**	752	751, 509> 273, 515>209	-	N.I.15	-	-

93	7.70	811.4339*	789.4230**	766	273, 765, 509> 241> 209	-	N.I.16	-	-
94	8.38	537.1557	539.1685	538	522> 507> 492, 479	-	N.I.17	-	-

[a] Retention time; [b] Molecular weight; [c] MS^n fragment patterns detected in the negative ion mode; [d] Tentative metabolites based on VIP analysis with a cut-off value of 0.7 and *p*-value < 0.05; [e] Reference; * $[M+HCOO]^-$; ** $[M+Na]^+$
